# Association between BMI and osteoporotic fractures at different sites in Chinese women: a case-control retrospective study in Changsha

**DOI:** 10.1186/s12891-024-07271-x

**Published:** 2024-02-29

**Authors:** Hong-Li Li, Yi Shen, Li-Hua Tan, Song-Bo Fu, Cong-Hui Guan, Dong-Hu Zhen, Hai-Hong Lv, Xi-Yu Wu, Xu-Lei Tang

**Affiliations:** 1 Department of Endocrinology, The First Hospital of Lanzhou University, No.1 DongGang West Road, Lanzhou, Gansu 730000 PR China; 2 Department of Metabolism and Endocrinology, and Hunan Provincial Key Laboratory for Metabolic Bone Diseases, National Clinical Research Center for Metabolic Diseases, The Second Xiangya Hospital, Central South University, No.139 Middle Renmin Road, Changsha, Hunan 410011 PR China; 3 Department of Orthopaedics, The Second Xiangya Hospital, Central South University, No.139 Middle Renmin Road, Changsha, Hunan 410011 PR China; 4 Department of Radiology, The Second Xiangya Hospital, Central South University, No.139 Middle Renmin Road, Changsha, Hunan 410011 PR China

**Keywords:** BMI, Osteoporotic fracture, Fracture risk, Bone mineral density, Female

## Abstract

**Background:**

Osteoporotic fractures are a growing problem in an aging society. The association between body mass index (BMI) and osteoporotic fractures varies by fracture site and ethnicity. Limited knowledge exists regarding this association in native Chinese, particularly utilizing local databases as reference sources.

**Objective:**

To investigate the association between BMI and osteoporotic fractures at different sites in Chinese women.

**Methods:**

Three thousand ninety-eight female patients with radiographic fractures and 3098 age- and sex-matched healthy controls without fractures were included in the study. Both of them underwent assessment using dual-energy X-ray absorptiometry (DXA), with BMD measurements calculated using our own BMD reference database. Participants were classified into underweight (BMI < 18.5 kg/m^2^), normal weight (18.5 ≤ BMI < 24.0 kg/m^2^), overweight (24 ≤ BMI < 28 kg/m^2^) and obese (BMI ≥ 28 kg/m^2^) according to the Chinese BMI classification standard.

**Results:**

There were 2296 (74.1%) vertebral fractures, 374 (12.1%) femoral neck fractures, and 428 (13.8%) other types of fractures in the case group. Bone mineral density (BMD) was almost lower in the fracture groups compared to the control groups (*p* = 0.048 to < 0.001). Compared with normal weight, underweight had a protective effect on total [odds ratio (OR) = 0.61; 95% confidence interval (CI), 0.49 –0.75; *P*< 0.001], and lumbar fractures (OR = 0.52; 95% CI, 0.41 – 0.67; *P* < 0.001), while obesity was associated with an increased risk for total (OR = 2.26; 95% CI, 1.85 – 2.76; *P* < 0.001), lumbar (OR = 2.17; 95% CI, 1.72 – 2.73; *P* < 0.001), and femoral neck fractures (OR = 4.08; 95% CI, 2.18 – 7.63; *P* < 0.001). Non-linear associations were observed between BMI and fractures: A J-curve for total, lumbar, and femoral neck fractures, and no statistical change for other types of fractures. Underweight was found to be a risk factor for other types of fracturess after adjusting for BMD (OR = 2.29; 95% CI, 1.09 – 4.80; *P* < 0.001). Osteoporosis and osteopenia were identified as risk factors for almost all sites of fracture when compared to normal bone mass.

**Conclusions:**

Underweight has a protective effect on total and lumbar spine fractures in Chinese women, while obesity poses a risk factor for total, lumbar, and femoral neck fractures. The effect of BMI on fractures may be mainly mediated by BMD.

## Background

Osteoporosis is a systemic skeletal disorder characterized by reduced bone mass, deteriorating microstructure of bone tissue, and diminished bone strength, resulting in increased vulnerability to fractures and fragility [1]. The lifetime risk of fracture for women and men over 50 years of age is 46.4% and 22.4%, respectively [2]. Osteoporotic fractures are defined as low-injury fracture, i.e. falls from standing height or lower, or without any fall [3, 4]. The detrimental consequences of osteoporotic fractures can be categorized into three main domains: disability, mortality, and escalated healthcare expenses [2, 5–22], excess mortality after a hip fracture ranges from 6% to 37% [5]. Preventive measures can be taken to reduce the occurrence of osteoporotic fractures by identifying high-risk groups early on and initiating appropriate treatment [6–9]. Safe and effective medications are available to reduce the risk of fractures [10].

Significant regional and ethnic differences in fracture rates have been found (hip fracture rates varied by a factor of more than 10) [6, 7, 11–14]. The prevalence of osteoporotic fractures in Chinese women and men was only 14.9% and 12.2%, respectively [15]. However, due to demographic shifts, it is projected that half of the world's osteoporotic fractures will occur in Asia, primarily in China, by 2050 [14]. Therefore, early assessment and prevention of fragility fractures are particularly crucial. Bone mineral density (BMD) plays a vital role in assessing fracture risk; however, accurate evaluation necessitates the identification and integration of additional risk factors other than BMD [6, 7, 9, 11, 16], including weight and body mass index (BMI) [7, 11–13, 16–22]. It has long been postulated that obesity confers protection against fractures due to the high BMD associated with obesity as well as the protective effect provided by hip soft tissue during falls [12, 17, 18]. Nevertheless, this perspective has faced challenges [7, 11–13, 16–26]. A meta-analysis conducted on postmenopausal women with osteoporotic fractures revealed that 27.7% of women with fractures had a BMI ≥ 30 kg/m^2^ [11]. Women with a BMI ranging from 25.0 to 27.4 kg/m^2^ exhibited the lowest incidence of hip fracture [13]. Findings from the Global Longitudinal Study of Osteoporosis in Women (GLOW) indicated that obesity served as a risk factor for ankle and thigh fractures but acted protectively against wrist fractures [12]. Studies have demonstrated site-specific variations in the impact of BMI on fractures [7, 12, 16–19].

There is limited research exploring the association between BMI and fracture risk in Asian populations. Relevant studies conducted in Japan and South Korea both suggested an increased fracture risk associated with underweight or obesity among women [7, 13, 18], indicating a non-linear relationship between BMI and fractures at specific sites [7, 13, 18]. The association between BMI and osteoporotic fractures at different sites in Chinese women remains unknown. Therefore, we conducted the case-control study to investigate this association in Chinese women.

## Methods and materials

### Participants

The retrospective study was conducted at Xiangya Second Hospital, Central South University, Changsha, China, from March 2011 to November 2021. This study was approved by the Ethics Committee of the Second Xiangya Hospital, affiliated to the Central South University, and each participant signed an informed consent form.

### BMD measurement

BMD of the lumbar spine (L1-L4), femoral neck (left), and total hip were measured by fan-beam dual-energy X-ray (DXA) absorptiometry (Hologic DelphiA; Hologic, Bedford, MA, USA). The right hip was measured only if the patient had a fracture or artificial replacement of the left femoral neck. The operator is the technician assigned by the hospital to do the examination full-time. The precision of BMD measurement, determined by the Root Mean Square Coefficient of Variation (RMSCV) method, were 0.86% for the spine, 1.17% for the femoral neck and 0.83% for the total hip in 33 subjects with two replicates. Long-term (>17 years) daily CV measurements of conventional quality control body models were < 0.45% as measured by DXA bone densitometry. Sex-specific BMD T-values for the lumbar spine, femoral neck, and total hip were calculated using our own BMD reference database [27]. According to the World Health Organization (WHO) definition [28], T-values > –1.0 indicate normal BMD; T-values ≤ –1.0 to > –2.5 and ≤ –2.5 are classified as osteopenia and osteoporosis, respectively.

### Inclusion criteria, exclusion criteria and data collection

Inclusion criteria: Cases: (1) Women aged ≥ 40 years old who volunteered to participate and signed informed consent; (2) Patients with osteoporotic fracture diagnosed according to clinical manifestations and imaging data; (3) Combined with confirmation by radiologists; vertebral fractures were confirmed on lateral vertebral radiographs using a semi-quantitative method [29]. Controls: (1) Local healthy individuals from the reference population, a database previously established by us [27]; (2) Matched 1:1 for sex and age with cases.

Exclusion criteria: Cases: (1) Traumatic fractures (such as car accidents or falls from above height); (2) Local pathological fractures caused by cancer or metastatic fractures; (3) Fracture sites not covered in this study; (4) All vertebrae were filled with artificial bone cement or contained an installed metal scaffold; (5) Had a bilateral femoral neck or hip fracture. Controls: (1) Had history of low- or high-injury fractures; (2) Osteosclerosis, bone fluorosis, or abnormal increase in bone density.

If part of the vertebral body of patients with fractures was filled with artificial bone cement or contained an installed metal scaffold, these lumbar vertebrae were excluded from the analysis, and the remaining vertebrae could still be used for subsequent calculations.

### BMI classification

Body mass index (BMI) is calculated as weight (kg) / height^2^ (m^2^). According to the BMI classification standard of overweight and obese adults in China [30], BMI < 18.5 kg/m^2^ was considered underweight, BMI = 18.5–23.9 kg/m^2^ was considered normal weight, BMI = 24.0–27.9 kg/m^2^ was considered overweight, and BMI ≥ 28.0 kg/m^2^ was considered obese.

### Statistical analysis

Data analysis was performed using SPSS V23.0 (SPSS Inc., Chicago, IL, USA) for Windows. The mean ± standard deviation (SD) is used to present the basic characteristics of the data. One-way analysis of variance (ANOVA) (The comparison between the case and control groups involves inter-group analysis, for which both ANOVA and independent sample T-test can be employed, yielding identical outcomes.) was used to compare age, age at menopause (AM), years since menopause (YSM), height, weight, BMI and BMD between the case and control groups. The odds ratio (OR) and 95% confidence interval (CI) were calculated using univariate and multivariate binary logistic analysis. In the multivariate analysis, we adjusted for BMD. The association between BMI and the risk of osteoporotic fractures at different sites was analysed using restricted cubic spline plots. The chi-squared test was used to compare the prevalence of osteoporosis and osteopenia between the case and control groups and within different skeletal sites. *P* < 0.05 indicated that the effect was statistically significant.

## Results

### Baseline characteristics

A total of 3098 patients with osteoporotic fractures aged 40–94 years consisted of the case group. There were 2296 (74.1%) vertebral fractures, 374 (12.1%) femoral neck fractures, and 428 (13.8%) other fractures (including forearm, shoulder, wrist, tibia, humerus, and rib fractures) in the case group, with a mean age (± SD) of 67.0 ± 8.71 years (Table 1). The BMDs in the case groups were were generally lower than those in the control groups (*P* < 0.05), except for the lumbar spine in the other fracture types. BMDs remained lower in the case group than those in the control group in BMI classification (Table 2). The proportions of low and normal weight patients were higher in the case groups compared with the control group (9.3% vs 4.9%, 56.2% vs 49.4%, *P* < 0.05), while the proportions of overweight and obese patients were lower (29.2% vs 35.3%, 5.3% vs 10.4%, *P* < 0.05). In the obese group, AM, height and weight were significantly lower in the case group than in the control group, but there was no significant difference in BMI. In the overweight group, AM, height, weight and BMI were significantly lower in the case group than in the control group. In the normal weight group, AM, height, weight and BMI were significantly lower in the case group than in the control group.

**Table 1:** Comparison of variables of clinical fractures according to fracture sites

**Variable**	**Case(TOTAL)**	**Control**	**Case(VF)**	**Control**	**Case(FF)**	**Control**	**Case(OF)**	**Control**
**n (%)**	3098(100)	3098(100)	2296(74.1)	2296(74.1)	374(12.1)	374(12.1)	428(13.8)	428(13.8)
**Age(years)**	67.0±8.71	67.0±8.72	67.0±8.40	67.0±8.40	70.2±9.32	70.2±9.32	64.0±8.78	64.0±8.78
**AM(years)**	48.3±3.70^a^	49.1±3.78	48.3±3.55^b^	49.1±3.78	48.6±3.65^b^	49.2±3.71	48.2±4.42^b^	49.0±3.84
**YSM(years)**	19.0±8.78^b^	18.4±8.96	19.0±8.50^a^	18.3±8.71	21.8±9.32	21.4±9.87	16.4±9.04	16.2±8.72
**Height(cm)**	150.9±6.39^a^	152.2± 5.21	150.2±6.27^a^	152.2±5.21	152.0±6.71	151.7±5.37	153.4±5.98^b^	153.4±5.05
**Weight(kg)**	51.9±8.53^a^	55.2±8.78	51.3±8.61^a^	55.3±8.82	52.2±7.84^a^	54.8±8.99	55.0±7.96	54.9±8.32
**BMI(kg/m**^**2**^**)**	22.8±3.28^a^	23.8±3.43	22.7±3.37^a^	23.8±3.44	22.6±3.07^a^	23.8±3.59	23.3±2.96	23.6±3.24
**PA-BMD(g/cm**^**2**^**)**	0.632±0.114^a^	0.754±0.139	0.613±0.108^a^	0.762±0.134	0.661±0.115^a^	0.753±0.143	0.706±0.111	0.715±0.152
**FN-BMD(g/cm**^**2**^**)**	0.514± 0.095^a^	0.618± 0.112	0.509±0.094^a^	0.614±0.109	0.495±0.091^a^	0.600±0.112	0.559±0.094^a^	0.659±0.122
**Hip-BMD(g/cm**^**2**^**)**	0.593± 0.116^a^	0.695±0.129	0.587±0.115^a^	0.691±0.126	0.572±0.108^a^	0.671±0.132	0.646±0.113^a^	0.735±0.132

Data are means ± standard deviations (SDs)

*VF* vertebral fracture, *FF* femoral neck fracture, *OF* other types fractures *AM*, age at menopause, *YSM* years since menopause, *BMI* body mass index *PA* posteroanterior spine *BMD* bone mineral density; *FN* femoral neck, Hip total hip

^a^*p* < 0.001 compared with controls

^b^*p* = 0.048–0.005 compared with controls

**Table 2:** Comparison of variables of clinical fractures according to body mass index categories

**Variable**	**UW**		**NW**		**OW**		**OB**	
	**Case**	**Control**	**Case**	**Control**	**Case**	**Control**	**Case**	**Control**
**n (%)**	288(9.3)^a^	153(4.9)	1742(56.2)^a^	1528(49.4)	905(29.2)^a^	1094(35.3)	163(5.3)^a^	323(10.4)
**Age(years)**	68.0±9.68	69.7±9.51	66.8±8.88	68.6±8.89	67.0±8.18	66.9±8.38	67.1±8.02	68.0±8.34
**AM(years)**	48.1±3.44	48.7±3.96	48.3±3.69^a^	49.2±3.67	48.5±3.80^b^	49.1±3.83	48.0±3.63^b^	48.9±4.02
**YSM(years)**	20.2±9.45	21.5±9.86	18.9±8.84^b^	17.9±9.12	18.8±8.39	18.4±8.55	19.3±8.7	19.6±8.71
**Height(cm)**	151.1±6.58	151.6±4.52	151.0±6.29^a^	152.2±5.22	150.9±6.13^a^	152.3±5.35	149.0±8.06^a^	151.7±4.95
**Weight(kg)**	39.3±4.37	39.4±3.51	49.2±5.56^a^	50.4±4.95	58.2±5.14^a^	59.8±4.79	67.0±7.87^a^	69.4±6.44
**BMI(kg/m**^**2**^**)**	17.2±1.12	17.1±1.06	21.5±1.49^a^	21.7±1.45	25.5±1.04^a^	25.7±1.10	30.1±2.18	30.1±2.10
**PA-BMD(g/cm**^**2**^**)**	0.562±0.107^a^	0.650±0.152	0.621±0.109^a^	0.728±0.134	0.665±0.110^a^	0.785±0.130	0.689±0.122^a^	0.822±0.126
**FN-BMD(g/cm**^**2**^**)**	0.454±0.090^a^	0.521±0.104	0.506±0.093^a^	0.596±0.107	0.540±0.087^a^	0.642±0.105	0.558±0.098^a^	0.686±0.103
**Hip-BMD(g/cm**^**2**^**)**	0.511±0.105^a^	0.581±0.133	0.580±0.111^a^	0.667±0.124	0.632±0.108^a^	0.725±0.116	0.660±0.122^a^	0.779±0.128

Data are means ± standard deviations (SDs)

*AM* age at menopause, *YSM* years since menopause, *BMI* body mass index, *PA* posteroanterior spine, *BMD* bone mineral density, *FN* femoral neck, Hip, total hip, *UW* underweight, *NW* normal weight, *OW* overweight, *OB* obesity

^a^*p* < 0.001 compared with control

^b^*p* = 0.030–0.001 compared with control

### Association of BMI and fractures

**Fig. 1:**
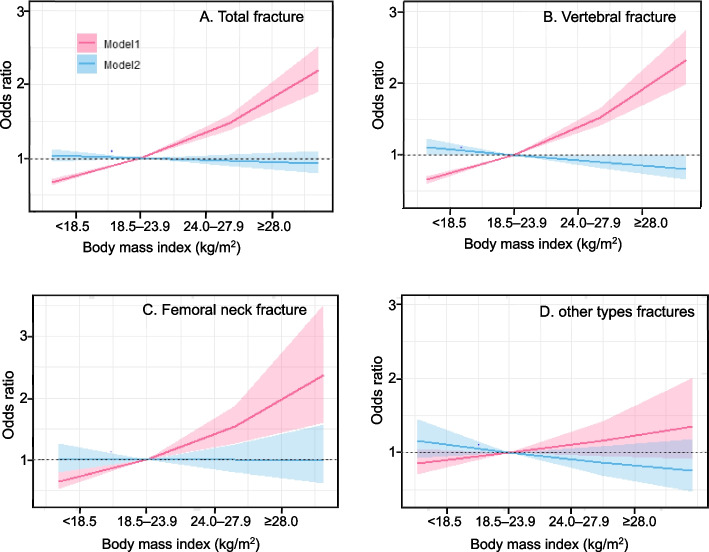
ORs for fracture risk by fracture site ORs, odds ratios Model 1: Crude, Model 2: Adjusted for BMD

Non-linear associations between BMI and fracture risk were observed: as BMI increased, the risk of fracture for total, lumbar, and femoral neck fractures increased, generally resembling a J curve. However, no statistically significant association was found between BMI and other types of fractures (Fig. 1). Compared with normal weight, underweight was a protective factor for total and lumbar fractures, decreasing the risk of fracture by 39% and 48% respectively. Overweight increased the risk of total and lumbar fractures by 38% and 43%, respectively, and obesity increased the risk of total, lumbar and femoral neck fractures by 126%, 117% and 308%, respectively (Fig. 1). The risk of fracture increased approximately 1.09 (95% CI, 1.08 – 1.11), 1.10 (95% CI, 1.08 – 1.12), and 1.11 (95% CI, 1.06 – 1.16) times for each unit increase in BMI for total, lumbar, and femoral neck fractures, respectively. After adjustment for BMD, the impact of BMI on most fracture sites became insignificant as BMI increased. However, adjusted underweight became a risk factor for the other fracture type.

Age stratification was performed for all fracture groups to account for the effect of age on fractures (Table 3). In comparison to normal weight, underweight was found to be a protective factor against fractures and obesity was identified as a risk factor for fractures in all age groups, while overweight posed a risk factor for fractures in those aged ≥ 60 years for total fractures. Concerning lumbar fractures, underweight demonstrated a protective factor in all age groups, whereas overweight and obesity were associated with an increased risk of fractures for those aged ≥ 60 years. For femoral neck fractures, underweight acted as a protective factor in people aged 40-59 years, while obesity emerged as a risk factor among people aged ≥ 60 years. The fracture risks (OR) of obesity for the age groups of 40-59 years old, 60-69 years old, and ≥70 years old were 1.65, 2.25, and 2.60 for total fractures, respectively,1.58, 2.27, and 2.4 for lumbar fractures, respectively, and 1.29, 5.04, and 4.57, for femoral neck fractures, respectively. The increasing risk associated with being overweight for total and lumbar fractures was observed specifically among individuals aged 60-69 years (OR 1.41 and 1.40, respectively) and ≥70 years (OR 1.47 and 1.47, respectively). In terms of other fracture types, the relationship between BMI and fracture occurrence remained unclear across all ages. After adjusting for BMD following age stratification, all the correlations between BMI levels and fracture incidence disappeared.

**Table 3:** ORs for fracture risk by age according to BMI rang and fracture sites

**Sites**	**Age group**	**N**	UW		**NW**	**OW**		**OB**	
			**Model 1**	Model 2		**Model 1**	**Model 2**	**Model 1**	**Model 2**
**Total**	40-59years	609	**0.44(0.27–0.72)**	0.74(0.41–1.34)	1(Reference)	1.28(0.99–1.66)	0.92(0.67–1.26)	**1.65(1.03–2.63)**	0.87(0.49–1.55)
	60-69years	1203	**0.56(0.38–0.81)**	0.95(0.63–1.44)	1(Reference)	**1.41(1.19–1.69)**	0.90(0.73–1.10)	**2.25(1.64–3.10)**	1.15(0.80–1.65)
	≥70years	1286	**0.72(0.54–0.97)**	1.07(0.78–1.48)	1(Reference)	**1.40(1.17–1.66)**	0.93(0.76–1.13)	**2.60(1.91–3.54)**	1.30(0.92–1.83)
**VF**	40-59years	433	**0.47(0.27–0.82)**	0.88(0.43–1.80)	1(Reference)	1.28(0.94–1.73)	0.80(0.54–1.20)	1.58(0.94–2.69)	0.66(0.33–1.31)
	60-69years	917	**0.50(0.33–0.77)**	0.97(0.59–1.60)	1(Reference)	**1.47(1.20–1.80)**	0.82(0.64–1.06)	**2.27(1.58–3.24)**	0.82(0.52–1.26)
	≥70years	946	**0.56(0.40–0.79)**	1.01(0.68–1.51)	1(Reference)	**1.47(1.20–1.80)**	0.80(0.63–1.02)	**2.40(1.66–3.46)**	0.87(0.57–1.34)
**FF**	40-59years	45	**0.10(0.01–0.81)**	0.42(0.04–4.93)	1(Reference)	1.15(0.42–3.18)	0.32(0.07–1.54)	1.29(0.20-8.37)	0.14(0.01–2.50)
	60-69years	120	0.63(0.20–1.95)	0.61(0.14–2.63)	1(Reference)	1.58(0.91–2.75)	0.80(0.40–1.64)	**5.04(1.35–18.76)**	1.11(0.21–5.91)
	≥70years	209	1.36(0.68–2.72)	1.69(0.82–3.49)	1(Reference)	1.21(0.77–1.89)	0.85(0.52–1.40)	**4.57(2.09–10.02)**	1.89(0.78–4.57)
**OF**	40-59years	262	0.74(0.20–2.73)	0.81(0.19–3.49)	1(Reference)	1.35(0.77–2.34)	1.18(0.63–2.20)	2.23(0.65–7.68)	1.80(0.42–7.72)
	60-69years	332	1.79(0.41–7.73)	1.18(0.25–5.51)	1(Reference)	1.09(0.69–1.72)	1.25(0.78–2.01)	1.40(0.58–3.36)	1.59(0.65–3.88)
	≥70years	262	1.66(0.60–4.62)	1.42(0.49–4.11)	1(Reference)	1.22(0.71–2.08)	1.45(0.82–2.55)	1.81(0.73–4.46)	2.01(0.78–5.18)

Model 1 Crude, Model 2 Adjusted for BMD

*ORs* odds ratios, *BMI*, body mass index, *VF* vertebral fracture, *FF* femoral neck fracture, *OF* other types fractures, *UW* underweight, *NW* normal weight, *OW* overweight, *OB* obesity

### Incidence of osteoporosis and its effect on fractures

The prevalence of osteoporosis was significantly higher in the case groups compared to the control groups, with lower rates of osteopenia and normal bone mass observed at most sites. Osteoporosis rates varied across different bone sites within the same fracture group, with the highest prevalence found in the lumbar spine for both case and control groups (Table 4).

**Table 4:** Number and rates of osteoporosis, osteopenia and normal BMD using gender specific T-scores according to fracture sites

**Fracture** **site**	**Skeletal site**	**Case**			**Control**		
		**Osteoporosis** **n (%)**	**Osteopenia** **n (%)**	**NBMD** **n (%)**	**Osteoporosis** **n (%)**	**Osteopenia** **n (%)**	**NBMD** **n (%)**
**Total**	PA	2691 (86.9)^abc^	379 (12.2)^ac^	28 (0.9)^abc^	1596 (51.5)^bc^	1205 (38.9)^b^	297 (9.6)^bc^
	FN	2287 (73.8)^ac^	737 (23.8)^a^	74 (2.4)^ac^	1043 (33.7)^c^	1423 (45.9)^c^	632 (20.4)^c^
	Hip	1824 (58.9)^a^	1126 (36.3)^a^	148 (4.8)^a^	810 (26.1)	1629 (52.6)	659 (21.3)
**VF**	PA	2085 (90.8)^abc^	191 (8.3)^ab^	20 (0.9)^abc^	1112 (48.4)^bc^	929 (40.5)^bc^	255 (11.1)^bc^
	FN	1741 (75.8)^ac^	512 (22.3)^a^	43 (1.9)^ac^	802 (34.9)^c^	1060 (46.2)	434 (18.9)
	Hip	1408 (61.3)^a^	793 (34.5)^a^	95 (4.1)^a^	613 (26.7)	1220 (53.1)	463 (20.2)
**FF**	PA	308 (82.4)^ac^	58 (15.5)	8 (2.1)^a^	183 (49.0)^bc^	149 (39.8)	42 (11.2)^c^
	FN	303 (81.0)^ac^	67 (17.9)^a^	4 (1.1)^ac^	142 (38.0)	174 (46.5)	58 (15.5)
	Hip	244 (65.2)^a^	118 (31.6)^a^	12 (3.2)^a^	120 (32.1)	192 (51.3)	62 (16.6)
**OF**	PA	283 (66.1)^bc^	130 (30.4)^a^	15 (3.5)^ac^	261 (61.0)^bc^	127 (29.7)^bc^	40 (9.3)^bc^
	FN	243 (56.8)^ac^	158 (36.9)^a^	27 (6.3)^ac^	99 (23.1)	189 (44.2)	140 (32.7)
	Hip	172 (40.2)^a^	215 (50.2)^a^	41 (9.6)^a^	77 (18.0)	217 (50.7)	134 (31.3)

*VF* vertebral fracture, *FF* femoral neck fracture, *OF* other types fractures, *PA* posteroanterior spine, *FN* femoral neck, Hip, total hip, *NBMD* normal bone mineral density

^a^*p* = 0.005 to < 0.001 compared with control

^b^*p* = 0.019 to < 0.001 compared with femoral neck on same parameter

^c^*p* = 0.049 to < 0.001 compared with total hip on same parameter

**Fig. 2:**
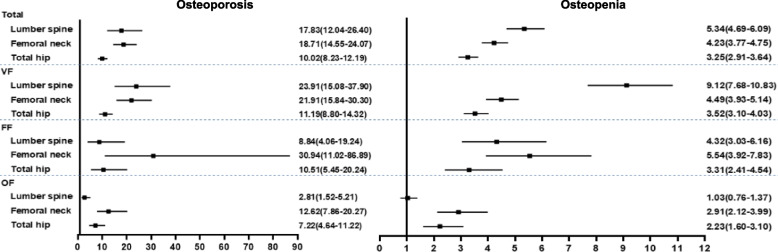
ORs for fracture risk of osteoporosis and osteopenia according to fracture sites. ORs, odds ratios; VF, vertebral fracture; FF, femoral neck fracture; OF, other types fractures

The effects of osteoporosis and osteopenia on fracture were calculated using normal bone mass as a reference (Fig. 2). Osteoporosis was identified as a significant risk factor for fractures at various bone sites across all fracture groups. Similarly, osteopenia also posed a risk factor at most sites, with the exception of the lumbar spine in other types of fractures. Furthermore, the ORs for osteoporosis and osteopenia varied among different bone sites within each fracture group, with significantly higher ORs observed for osteoporosis compared to those for osteopenia.

## Disscussion

We present the findings of a sex- and age-matched case-control retrospective study, wherein patients with clinical osteoporotic fractures were included as cases, while individuals without fractures served as controls. The associations between BMI and fracture risk were found to be complex. Underweight exhibited a protective factor against total and vertebral fractures; however, it became a risk factor for other types of fractures after adjusting for BMD. Overweight posed a risk for both total and vertebral fractures in individuals aged 60 years and older, demonstrating site- and age-specific associations between BMI and fracture risk. BMDs were significantly lower in the case groups compared to the control groups, irrespective of fracture site or BMI classification, suggesting that this may be an important contributing factor to fractures. Furthermore, after adjusting for BMD at most sites, the association between BMI and fractures disappeared, implying that this association is primarily mediated by BMD.

BMI has a strong correlation with body fat percentage and is largely independent of height [31]. Previous analyses have demonstrated that both overweight, obesity [7, 11, 13, 14, 16, 17, 19], and underweight [7, 13, 18, 22] may increase the risk of fractures at certain sites. Conversely, obesity may be protective against fractures at some sites [16, 17, 19, 22, 32]. Studies have shown that for every 1 kg/m^2^ increase in BMI, the risk ratio was 0.97 (95%CI=0.96-0.98) for osteoporotic fractures, and 0.93 (95%CI= 0.91-0.94) for hip fractures [32]. When included in the same regression model, BMI primarily reflects lean body mass while waist circumference predominantly indicates abdominal obesity; higher BMI was independently associated with a lower risk of lumbar fractures, suggesting that fat distribution plays an important role in predicting vertebral fractures [20]. Biopsy studies have confirmed that premenopausal women with central adiposity exhibit poorer bone quality and stiffness along with significantly lower bone formation [26]. Lean body mass has been shown to exert a greater influence on BMD compared to fat mass [23], moreover, the accumulation of visceral fat associated with obesity has been negatively correlated with bone strength [24, 26], and abdominal obesity is associated with increased hip fracture risks [25]. However, some studies have failed to establish any statistical association between BMI and vertebral fractures in women [21]. The effect of BMI on fractures at a given BMD level remains controversial, partly because the effect varies at different fracture sites [7, 12, 16–19]. It has been suggested by several studies that obesity alone may not confer protection against fractures; instead, the effects of physical strength, activity, body composition, muscle strength, and fat distribution on fractures should be further considered in this relationship [11, 14, 16, 19, 22, 23, 25, 26]. In addition, studies indicated that obese patients tend to have a poorer prognosis following fracture surgery [11, 12, 33]. Given the escalating prevalence of obesity in the population at large, further investigation is warranted to elucidate the pathogenesis of fractures in obese individuals and to identify appropriate prevention strategies.

Current studies on the association between BMI and fractures are mainly focus on Western populations, which typically have higher BMIs compared to Asian populations [11]. However, there is a lack of research specifically examining Asians, thus limiting the generalizability of previous findings to this population. According to the Chinese adult obesity standard (BMI ≥ 28.0kg/m^2^ is considered obese) [30], only 5.3% of female fracture patients in this study were classified as obese, significantly lower than the reported rate of 27.7% in Western populations [11], suggesting significant ethnic differences. The overall incidence of osteoporosis was 86.9% in our study, which shown distinct ethnic variation compared to previous studies, as well as differences in the incidence of osteoporosis among patients with osteoporotic fractures. Therefore, it is important to recognize the exist of variation between Asian and European populations [31]. Therefore, data from Asian populations are imperative to classify BMI and further elucidate the association between BMI and fracture risk. Our study revealed a non-linear relationship between BMI and fractures: fracture risk increased with higher BMI for total, lumbar and femoral neck fractures resembling a J-curve, however, no statistically significant change was observed for other fracture types. The direct associations between BMI and fracture risk depend on the fracture site.

Osteoporosis and fractures are more prevalent in women. The literature indicates that 44% of nonvertebral fractures and 64% of hip fractures occur in women with osteoporosis, compared to approximately 21% and 39%, respectively, in men [34] suggesting a greater impact of BMD on female fractures than on male fractures. BMD is the primary determinant influencing fracture occurrence. After adjustment for BMD, the influence of BMI on fracture risk in women attenuated or eliminated, as our research demonstrated, indicating that BMI did not exert an independent influence on most fracture sites, mainly mediated on BMD. The BMDs in the case groups were significantly lower than those in the control groups, regardless of BMI classifications, suggesting that low bone mineral density plays a crucial role in increasing fracture risk. In terms of osteoporosis classifications (Fig. 2), the ORs for lumbar, femoral neck, and total hip osteoporosis were 3.34, 4.42, and 3.08 times higher than those for osteopenia, respectively. Different bone sites within each fracture group displayed varying incidence of osteoporosis and osteopenia along with different ORs for fracture risk; thus indicating that the effect of BMD varies depending on the specific fracture site. Furthermore, it can be inferred that the influence of BMI on fractures primarily operated by BMD, which may account for these observed disparities between BMI levels and fracture risk. Other measures (such as height, weight, age at menopause, years since menopause, etc.) exhibited significant variations between case and control groups in different fracture sites and different BMI classifications, suggesting that these variations had an impact on fractures.

Taking ethnicity, sex, age, fracture site and BMI classification into consideration may enhance the accuracy of fracture risk assessment. The disparities between our findings and previous research may be attributed to several factors. Firstly, it is possible that the increase in BMD with increasing BMI is less pronounced in Asians compared to European populations. This could be due to the fact that Asians generally have lower lean body mass and higher levels of body fat, particularly visceral adipose tissue, when compared to Caucasians. Additionally, the protective effect of fat around the legs and hips on fractures appears to be reduced in Asian women [31]. Therefore, we hypothesize that the relationship between BMD changes and increasing BMI among obese Asians differs from Western populations. Secondly, Westerners and Asians exhibit varying degree of metabolic disturbances associated with fat distribution at a given BMI [7, 19, 31], which can potentially affect fracture risk. Our research not only demonstrates the non-linear and site-specific relationship between BMI and fragility fractures in native Chinese individuals, but also contributes data collection on the association of BMI and fragility fracture in certain southern regions, which is a meaningful contribution to construct the epidemiologic data for Chinese. This is the major contribution of our work.

This study has some limitations. First, it is not a multi-centre study, and therefore its findings may only be representative of the selected population and its surrounding area. To eliminate geographical differences, larger multi-center studies are required. Second, this paper aimed to elucidate the associations between BMI and fracture sites, due to the limited number of samples, not all clinically visible fracture sites were included in the analysis, and the number of certain fracture sites was small, so certain fracture site combinations were made during statistical analysis which might not fully address the issue at hand. The third limitation pertains to potential underestimation of height measurements in patients with vertebral fractures, particularly multiple vertebral compression fractures, thereby impacting the accuracy of BMI calculations. BMI is not measured directly, but estimated using a formula related to the subject's height and weight, which may not accurately reflect body composition, and could potentially introduce bias. Additionally, due to the retrospective nature of the study, we were unable to include other variables that may affect fracture risk, such as sex, previous osteoporotic fractures, medication use, long-term glucocorticoid use, history of falls, parental hip fracture, long-term smoking, chronic alcohol use, rheumatoid arthritis, dementia, comorbidities, and various types of secondary osteoporosis. Therefore, it is imperative to conduct large prospective studies in order to control for these confounding factors.

In conclusion, our study has demonstrated site-specific variations in the relationship between BMI and osteoporotic fractures. The associations between fracture risk and BMI follow a J-shaped curve for total, lumbar, and femoral neck fractures. However, no statistically significant difference is found for other types of fractures. Underweight is associated with decreased risk of total and hip fractures, while overweight and obesity increase the risk of these same fractures. The effect of BMI on fracture varies by site and is primarily mediated by BMD. Both osteoporosis and osteopenia are risk factors for fractures, and osteoporosis is more significant.

## Data Availability

All data analyzed during this study are included in this published article.

## Abbreviations

BMD: Bone mineral density
DXA: Dual-energy X-ray absorptiometry
OR: Odds ratio
PA: Posteroanterior
RMSCV: Root-mean-square coefficient of variation
WHO: World Health Organization
CI: Confidence interval
PA-BMD: Posteroanterior (PA) spine bone mineral density
